# Recent Progress in Carbon-Based Buffer Layers for Polymer Solar Cells

**DOI:** 10.3390/polym11111858

**Published:** 2019-11-11

**Authors:** Thang Phan Nguyen, Dang Le Tri Nguyen, Van-Huy Nguyen, Thu-Ha Le, Dai-Viet N. Vo, Quang Viet Ly, Soo Young Kim, Quyet Van Le

**Affiliations:** 1 Laboratory of Advanced Materials Chemistry, Advanced Institute of Materials Science, Ton Duc Thang University, Ho Chi Minh City 700000, Vietnam; nguyenphanthang@tdtu.edu.vn; 2 Faculty of Applied Sciences, Ton Duc Thang University, Ho Chi Minh City 700000, Vietnam; 3 Institute of Research and Development, Duy Tan University, Da Nang 550000, Vietnam; dltnguyen@yahoo.com (D.L.T.N.); lyquangviet@yahoo.com (Q.V.L.); 4 Key Laboratory of Advanced Materials for Energy and Environmental Applications, Lac Hong University, Bien Hoa 810000, Vietnam; vhnguyen@lhu.edu.vn; 5 Faculty of Materials Technology, Ho Chi Minh City University of Technology (HCMUT), Vietnam National University–Ho Chi Minh City (VNU–HCM), 268 Ly Thuong Kiet, District 10, Ho Chi Minh City 700000, Viet Nam; lthuha2410@gmail.com; 6 Center of Excellence for Green Energy and Environmental Nanomaterials (CE@GrEEN), Nguyen Tat Thanh University, 300A Nguyen Tat Thanh, District 4, Ho Chi Minh City 755414, Vietnam; daivietvnn@yahoo.com; 7 State Key Laboratory of Separation Membrane and Membrane Processes, National Center for International Joint Research on Membrane Science and Technology, School of Materials Science and Engineering, Tianjin Polytechnic University, Tianjin 300387, China; 8 Department of Materials Science and Engineering, Korea University, 145 Anam-ro, Seongbuk-gu, Seoul 02841, Korea

**Keywords:** polymer solar cells (PSCs), buffer layer, carbon materials, work function

## Abstract

Carbon-based materials are promising candidates as charge transport layers in various optoelectronic devices and have been applied to enhance the performance and stability of such devices. In this paper, we provide an overview of the most contemporary strategies that use carbon-based materials including graphene, graphene oxide, carbon nanotubes, carbon quantum dots, and graphitic carbon nitride as buffer layers in polymer solar cells (PSCs). The crucial parameters that regulate the performance of carbon-based buffer layers are highlighted and discussed in detail. Furthermore, the performances of recently developed carbon-based materials as hole and electron transport layers in PSCs compared with those of commercially available hole/electron transport layers are evaluated. Finally, we elaborate on the remaining challenges and future directions for the development of carbon-based buffer layers to achieve high-efficiency and high-stability PSCs.

## 1. Introduction

**Figure 1:**
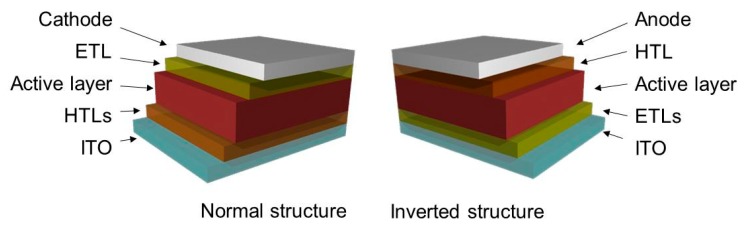
Structure of normal and inverted polymer solar cells (PSCs).

Currently, the demand for renewable energy resources has been increasing at an accelerated rate because of the depletion of sources of fossil energy, which have been predicted to be exhausted in the near future. Therefore, the search for new energy resources is critical and urgent. Solar energy has emerged as the most promising sustainable and clean alternative along with other green energy sources, e.g., wind energy, wave energy, and hydrogen fuel. Although silicon solar cells have been proved to be very efficient for transforming light to electricity, their successful application is still restrained by their relatively high production cost and intricate manufacturing process [1,2]. To overcome these challenges, many different new generations of solar cells have been proposed and investigated, including quantum dot solar cells [3,4,5], dye-sensitized solar cells [6,7], polymer solar cells (PSCs) [8,9,10,11,12], copper indium gallium selenide (CIGS) solar cells [13], and perovskite solar cells [14,15,16]. Among these, PSCs are considered important because of the numerous advantages such as high flexibility, less toxicity, and low production cost of the roll-to-roll fabrication process [17,18,19,20]. Nevertheless, several challenges need to be addressed before PSCs can be commercialized, such as their low power conversion efficiency and low stability [10,21]. In particular, PSC performance depends on numerous parameters such as device structure, electrodes, buffer layers, and active layers. In practice, PSCs can be fabricated into two different structures—regular and inverted structures—as shown in Figure 1. Besides electrodes and active layers, which are critical factors for determining PSC performance [10,22], buffers layers also have a profound impact on improving the charge transport from active layers to electrodes and device stability [23]. The most common polymer-based hole transport layer in PSCs is poly(3,4-ethylene dioxythiophene)-poly(styrene sulfonate) (PEDOT:PSS). However, PEDOT:PSS is a highly acidic and hygroscopic polymer that can easily absorb water in air and causes fatal damage to the active layers and anode [24,25,26]. Thus, for long-term device operation, PEDOT:PSS should be replaced by more stable hole transport layers.

Recently, carbon materials such as graphene, graphene oxide, carbon quantum dots, graphene quantum dots, carbon nanotubes, and graphitic carbon nitride have emerged as a new class of materials for energy conversion and storage devices such as supercapacitors [27,28], batteries [29,30], catalysts [31,32], and photovoltaic devices [33,34]. Because of their tunable optical and electrical properties, carbon materials have been thoroughly investigated as either hole transport layers or electron transport layers in solar cells [35]. Interestingly, the use of carbon materials as hole transport layers improves not only the light to electric current efficiency but also device stability [35]. Furthermore, most carbon material-based buffer layers are inexpensive and can be synthesized on a large scale [36,37,38,39,40]; thus, they are expected to be the most futuristic technology for traditional hole transport layers (HTLs) and electron transport layer (ETLs) toward commercialization.

In this review, we first focus on the structures and properties of carbon-based materials and their advantages as buffer layers. Next, we highlight the recent strategies for implementing these carbon-based materials as efficient buffer layers in PSCs. More precisely, recent approaches to modifying the optical and electrical properties of different carbon-based materials, including thermal treatment, chemical treatment, functionalization, doping, and compositional engineering toward high-performance PSCs, are summarized and discussed. Finally, the remaining challenges and directions for future development are included.

## 2. Structures and Properties

### 2.1. Graphene

**Figure 2:**
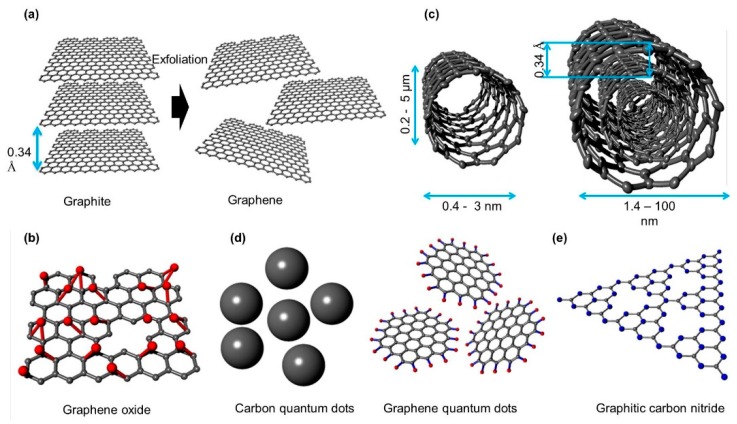
Structure of carbon materials (**a**) graphene, (**b**) graphene oxide (GO), (**c**) single-wall and multiple-wall carbon nanotubes, (**d**) carbon quantum dots and graphene quantum dots, and (**e**) graphitic carbon nitride.

Graphene is a monolayer, 2-dimensional carbon network organized in a honeycomb lattice with sp^2^ hybridization [41] and can be obtained through exfoliation of graphite [42,43] or metal-catalyzed chemical vapor deposition (structure shown in Figure 2a) [44]. In general, graphene exhibits a high in-plane electrical conductivity exceeding 1000 S m^−1^ [45], a high thermal conductivity of ~4000–5000 W m^−1^ k^−1^ [46,47], high optical transmittance of 98% [48], and large specific surface area of 2630 m^3^ g^−1^ [49]. In addition, graphene displays excellent mechanical properties with a tensile strength of ~130 GPa and Young’s modulus of 1 TPa [50]. These intrinsic properties suggest that graphene can be a promising candidate for applications in flexible electronic and optoelectronic devices [51]. Specifically, graphene has been demonstrated to be successful potential electrodes and buffer layers in PSCs. In addition to its good conductivity and transparency, work function is another factor that determines the performance of buffer layers in optoelectronic devices. Briefly, high work-function buffer layers can be employed as hole transport layers, whereas low work-function buffer layers can be utilized as electron transport layers. Because the work function of pristine graphene is relatively low (~4.2 eV), it can be only used as an electron transport material in photovoltaic devices [52,53]. However, it can be utilized as HTLs by forming a composite structure with other HTLs such as PEDOT:PSS, MoO_3_, and WO_3_.

### 2.2. Graphene Oxide

**Figure 3:**
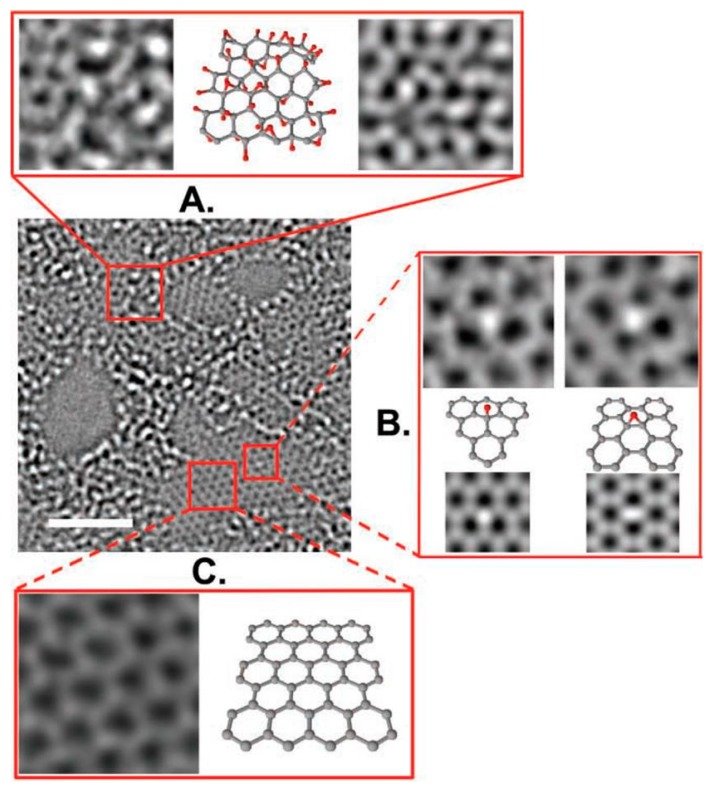
Aberration-corrected TEM image of a single sheet of suspended GO. The scale bar is 2 nm. Expansion (**A**) shows, from left to right, a 1-nm^2^ enlarged oxidized region of the material, then the proposed possible atomic structure of this region with carbon atoms in gray and oxygen atoms in red, and finally the average of a simulated TEM image of the proposed structure and a simulated TEM image of another structure where the position of oxidative functionalities has been changed. Expansion (**B**) focuses on the white spot on the graphitic region. This spot moved along the graphitic region, but remained stationary for 3 frames (6 s) at a hydroxyl position (left portion of expansion (**B**)) and for 7 frames (14 s) at a (1,2) epoxy position (right portion of expansion (**B**)). The ball-and-stick figures below the microscopy images represent the proposed atomic structure for such functionalities. The simulated TEM image for the suggested structure agrees well with the TEM data. Expansion (**C**) shows a 1 nm^2^ graphitic portion from the exit plane wave reconstruction of the focal series of GO and the atomic structure of this region. Reproduced with permission [57].

Graphene oxide (GO) is produced from the oxidation of graphene via reacting with a strong oxidant or acid which generally comprises numerous functional groups such as hydroxyl, epoxy, carbonyl phenol, lactone, quinine, and carboxyl groups (Figure 2b) [54,55]. Because the lattice structure of graphene is damaged and distorted upon oxidation, the structures and properties vary depending on the conditions of fabrication [54,55]. For more in-depth understanding, many different structure models have been proposed and investigated such as those by Hofmann and Holst, Ruess, Scholz and Boehm, Nakajima and Matsuo, and Left and Klinowski [56]. However, the actual structure of GO was not identified until 2010 when Erickson et al. reported a vivid illustration of the GO structure using high-resolution transmission electron microscopy (HR-TEM) [57]. As displayed in Figure 3, GO comprises three distinct areas—a graphitic region, holes, and a distorted region. Among these regions, the graphitic region was presumed to be the unreacted basal plane of graphene, whereas holes were created when graphene was exfoliated and oxidized, releasing CO and CO_2_ gases. Furthermore, the distorted region is postulated to originate from the highly distributed, oxygen-containing functional groups.

As mentioned above, the properties of GO are adjustable, primarily by controlling the functional groups and the degree of oxidation. In particular, GO with dominant sp^3^ hybridization has a wide bandgap and can thus be applied as an insulating material with a sheet resistant (Rs) of up to 12 ohm sq^−1^. Depending on the applications, the Rs of GO can be tuned via thermal or chemical reduction. Thus, GO can be tuned from an insulator to a semiconductor and ultimately a semi-metal. The increase in conductivity is attributed to the transformation of sp^3^ to sp^2^ hybridization [58]. In addition, the work function of GO can also be tuned from 5.72 to 4.43 eV upon treatment [58]. These properties indicate that GO can be employed in PSCs as either HTL or ETL; however, specific treatment should be applied.

### 2.3. Carbon Nanotube

**Figure 4:**
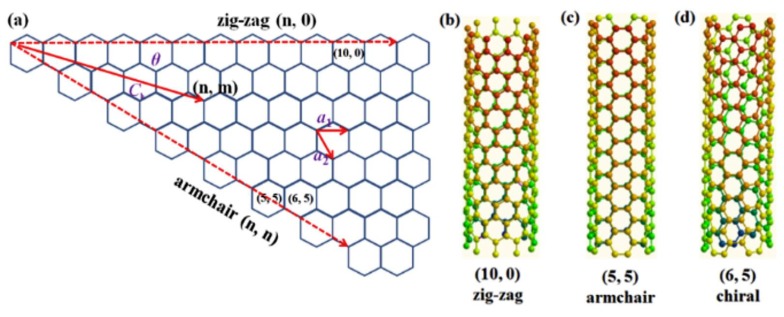
(**a**) Unrolled single layer graphene sheet showing the geometry of single-wall carbon nanotubes (SWCNTs). Examples of three types of nanotube (**b**) sidewalls—zig-zag, (**c**) armchair, and (**d**) chiral. Reproduced with permission [63].

Carbon nanotubes (CNTs) are graphene allotropes in which graphene sheets are rolled up to form a unique 1D cylindrical shape with sp^2^ hybridization [59]. Based on the number of layers (n), CNTs can be classified into single-wall CNTs (SWCNTs) (n = 1) and multiple wall CNTs (MWCNTs) (n ≥ 2) (Figure 2c) [60]. The diameter of SWCNTs ranges from 0.4 to 3 nm, while their length is up to a few million times of the diameter [61]. The diameter of MWCNTs, in contrast, has been estimated to be 1.4–100 nm, depending on the number of layers. The distance between each layer of MWCNTs is approximately 3.4 Å, which is similar to that of graphene in graphite [60,62]. The properties of CNTs are significantly influenced by the chiral vector, as shown in Figure 4 [62,63]. By controlling the direction of rolling, the properties of CNTs can be modulated between semiconductors and metals [64]. CNT based transparent conductive films exhibit an Rs of 208 and 24 Ω sq^−1^ with a transmittance of 90% and 83.4%, respectively. It is confirmed that there is a significant trade-off between electrical conductivity and transmittance [65]. To attain the best device performance, optimizing CNT thickness is essential. In addition, to determine the role of CNTs as buffer layers, the work function of CNTs needs to be verified. Interestingly, CNTs exhibit a much higher work function (~4.9–5.05 eV) compared with graphene (~4.2 eV), indicating that CNTs are potential HTLs rather than ETLs [66].

### 2.4. Carbon and Graphene Quantum Dots

Carbon quantum dots (CQDs) and graphene quantum dots (GQDs) are zero-dimensional carbon-based materials; however, they are two different allotropes with significant differences in their structures and properties (Figure 2d). In short, CQDs are quasi-spherical nanoparticles with a diameter under 10 nm that are formed either from crystalline graphite cores with sp^2^ hybridization or from amorphous carbon. In contrast, GQDs are fabricated from single or few graphene layers with a diameter of less than 10 nm. Basically, CQDs and GQDs are passivated with many complex functional groups such as carboxylate and hydroxylate derivatives that remain after fabrication. These functional groups largely affect the optical properties, electrical properties, and solubility of CQDs and GQDs [67]. Both CQDs and GQDs have been found to exhibit a pronounced quantum confinement effect, which enables them to emit light in a wide range of visible light depending on their diameters and surface/edge states [68,69,70]. GQDs inherit most of the properties of graphene and GO. Thus, they can also work well as buffer layers in PSCs. According to previous reports, the work function of GQDs is larger than 5 eV, suggesting that they are promising HTLs for PSCs [71]. Interestingly, the work function of GQDs can be reduced to 4–4.5 eV via alkaline metal doping, suggesting that they can also be utilized as ETLs with proper treatment [71].

### 2.5. Graphitic Carbon Nitride

Carbon nitride (C_3_N_4_) is a newly identified carbon-based material that has also drawn significant attention in recent years for application in photocatalysis and optoelectronic devices [72,73,74,75]. It has several allotropes, including graphitic C_3_N_4_ (g-C_3_N_4_), cubic-C_3_N_4_, pseudocubic-C_3_N_4_, a-C_3_N_4_, and b-C_3_N_4_ [76,77]. Among these, graphitic carbon nitride has been found to be the most stable structure comprising tri-s-triazine units connected to each other via tertiary amines (Figure 2e) [77]. Generally, g-C_3_N_4_ is a semiconductor with a wide bandgap of 2.7 eV with a high specific area of 2500 m^2^ g^−1^ [78]. Because of its wide bandgap, g-C_3_N_4_ displays an acceptable transmission that can be used in photovoltaic devices [79]. Furthermore, the work function of g-C_3_N_4_ was computed to be 4.65 eV and can be severely reduced by adding halogen dopants, suggesting that this material can function as ETLs in PSCs [80].

## 3. Application of Carbon Buffer Layers in PSCs

### 3.1. Graphene Buffer Layers

**Figure 5:**
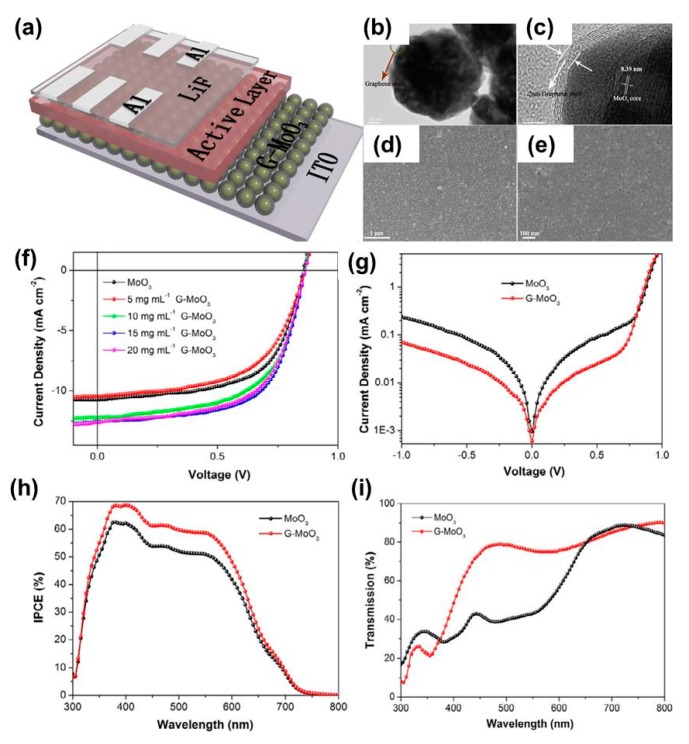
(**a**) Schematic diagram of PSCs showing the cross-sectional image of the device structure with the following layers: ITO/G-MoO_3_/PCDTBT:PC_71_BM/LiF/Al. (**b**) TEM image of G-MoO_3_ nanoparticles. (**c**) HRTEM image of MoO_3_ covered by graphene. (**d**) Low-magnification SEM of G-MoO_3_ films when applied to the surface of glass. (**e**) High-magnification SEM of G-MoO_3_ films when applied to the surface of glass. J–V curves of OSCs with MoO_3_ and hybrid G-MoO_3_ hole transport layers (HTLs) (**f**) under illumination and (**g**) in the dark. (**h**) IPCE spectra of PSCs with MoO_3_ and G-MoO_3_ HTLs. (**i**) The transmittance of MoO_3_ and G-MoO_3_ films. Reproduced with permission [81].

To date, the most reported applications of graphene in PSCs are related to transparent conductive electrodes rather than buffer layers. Nevertheless, several efforts have been made to introduce graphene as HTLs and ETLs by forming composites with other HTLs or ETLs. For example, Dang et al. found that the addition of graphene could enhance the hole extraction capability of MoO_3_-based HTLs by improving optical transmittance, electrical conductivity, and hole mobility [81]. Furthermore, the graphene-MoO_3_ composite exhibits a work function close to the highest occupied molecular orbital (HOMO) level of the organic active layers (Poly[N-9″-hepta-decanyl-2,7-carbazolealt-5,5-(4′,7′-di-2-thienyl-2′,1′,3′-benzothiadiazole)] (PCDTBT) [81]. Therefore, the power conversion efficiency of PSC can be improved by 17% compared with that of pristine MoO_3-_based devices. The device structure of PSCs, TEM images of G-MoO_3_, SEM images of G-MoO_3_ film, and device characteristics of G-MoO_3_-based PSCs are shown in Figure 5. Iakobson et al. developed a novel HTL by combining graphene nanosheets with polyaniline in the presence of poly(2-acrylamide-2-methyl-1-propanesulfonic acid) (PAMPSA) [82]. The composite HTLs exhibited good conductivity with a high work function of 5.27 eV, thus resulting in good PSC performance with a PCE of 2.9%.

### 3.2. Graphene Oxide Buffer Layers

**Figure 6:**
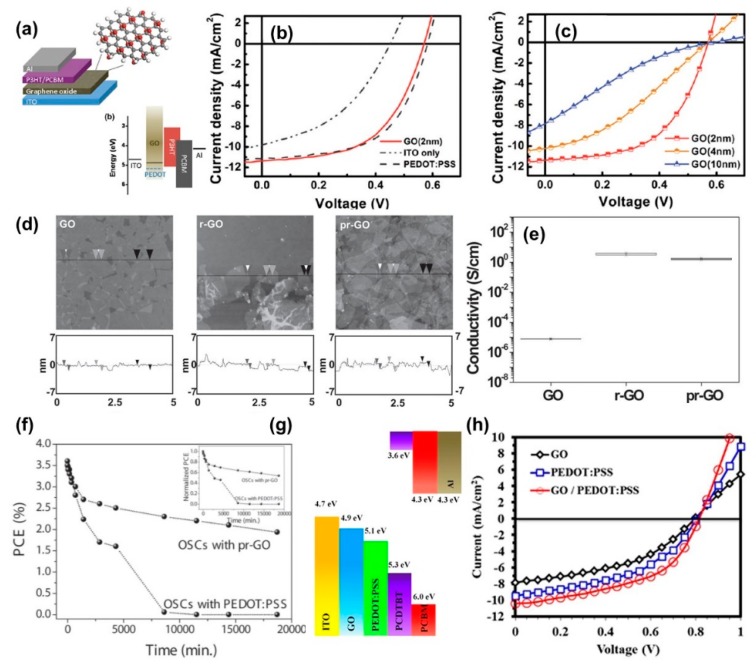
(**a**) Schematic and band diagram of a PSC device consisting of ITO/GO/P3HT: PCBM/Al. (**b**) Current–voltage characteristics of photovoltaic devices with no hole transport layer (curve labeled as ITO), with a 30-nm PEDOT: PSS layer and a 2-nm thick GO film. (**c**) Current–voltage characteristics of ITO/GO/P3HT: PCBM/Al devices with different GO thickness. All measurements were under simulated A.M. 1.5 illumination at 100 mW/cm^2^. Reproduced with permission [83]. (**d**) AFM images and (**e**) conductivity of spin-coated GO-film, r-GO film, and pr-GO film. Reproduced with permission [90]. (**f**,**g**) Schematic diagram and structure of PSCs using a GO/PEDOT: PSS double hole transport layer. (**h**) I–V curves of PSCs using GO, PEDOT: PSS, and GO/PEDOT: PSS as hole transport layers. Reproduced with permission [93].

In contrast to graphene, GO has received much more attention as HTLs because of its comparable work function and because it can be solution-processed on a large scale. The research on GO as HTLs has increased from 2010 onwards when Li et al. successfully demonstrated high-performance PSCs based on this material which is comparable to that of PEDOT:PSS [83]. Specifically, they fabricated GO nanosheets with a work function of 4.7 eV which is well-matched with the HOMO level of the organic donor in the bulk heterojunction active layer (poly(3-hexylthiophene, P3HT; Figure 6a). Consequently, GO-based PSCs exhibit a remarkable high PCE of 3.5%, which is about two-times higher than that of devices without HTL (PCE = 1.8%) and comparable with that of PEDOT: PSS based devices (PCE = 3.6%) (Figure 6b). Notably, the performance of GO-based HTLs significantly depends on GO thickness. Furthermore, when the GO thickness was increased from 2 to 10 nm, the PCE and fill factor of the polymers solar cells was drastically reduced from 3.5% and 54% to 0.9 and 19%, respectively (Figure 6c) [83]. The reduction in device performance is mainly attributed to the insulating nature of GO, which hinders the charge transport from active layers to the anode. Therefore, to attain better device performance, either the uniformity or the conductivity of the GO layer needs to be improved. In fact, the conductivity of the GO layer can be increased through chemical reduction or thermal reduction, resulting in reduced GO (rGO) [56]. However, the reduction of GO generally leads to a decrease in work function, thus forming an energy barrier between the active layers and HTLs. Therefore, the reduction process for GO should be carefully optimized to improve conductivity while maintaining the required work function. For example, Rafique et al. obtained a moderately reduced GO via UV–ozone treatment (UVO) and applied the GO as HTL in PSCs [84]. The moderate reduced GO showed a high work function of 4.9 eV with improved conductivity. Thus, 15-min UVO–GO-based PSCs (PCDTBT: [6,6]-Phenyl-C71-butyric acid methyl ester (PC_71_BM)) yield a PCE of 4.07%, which is two times higher than that of devices based on non-treated GO as HTLs. Similarly, Xia et al. improved the PCE of the PSC from 1.8% (pristine GO-HTLs) and 3.73% (PEDOT:PSS-HTLs) to 4.18% through a UVO-treatment on GO for 30 min [85]. The UVO-treated GO not only significantly improved the device performance but also device stability. Particularly, the UVO-treated GO based PSC maintained 90% of its original PCE after being kept for 40 days in an Ar-filled glovebox. Meanwhile, the PEDOT: PSS based device retained only 77% of its performance under the same storage time and condition. Apart from UVO reduction, thermal reduction is also a good approach that allows us to control the level of reduction precisely by controlling temperature and time. For instance, Jeon et al. performed post thermal reduction of GO films at different temperatures ranging from 150 to 250 and 350 °C and used the films as HTLs [86]. The best reduction temperature for GO as HTLs was found to be 250 °C (250-GO); at this temperature, a drastic increase in PEC of the solar cells from 1.47% (pristine GO-HTLs) to 3.89% was observed, which was higher than that of PEDOT:PSS based devices (PCE = 3.85%). The increase in PSC performance originated from the increase in electrical conductivity from 8 × 10^−6^ to 1.8 S m^−1^. These results are in perfect accordance with the results of a study conducted by Liu et al. [87]. At higher reduction temperature, the work function of GO reduces lower than the required work function for an HTL, thus deteriorating device performance [87]. Instead of merely using thermal reduction, Murray et al. retreated rGO with UVO to improve the work function, yielding PSC (Poly({4,8-bis[(2-ethylhexyl)oxy]benzo[1,2-b:4,5-b′]dithiophene-2,6-diyl}{3-fluoro-2-[(2-ethylhexyl)carbonyl]thieno[3,4b]thiophenediyl}) (PTB7):PC_71_BM) with a PCE of 7.39% and excellent stability [88]. More recently, Kwon et al. presented a new approach for the reduction of GO using an electron beam irradiation method. The performance of the ion beam treated GO as HTLs was found to be good [89]. However, this method included a complex process with expensive equipment and was costly. Thus, it was not developed further. Other than post-reduction on deposited GO film, GO can be reduced in a solution prior to film deposition. It should be noted that many functional groups will be removed upon reduction. Thus, the reduction of GO normally causes the aggregation of rGO in a solution which in turn results in a low-quality rGO film, thus lowering device performance [90]. To counter this problem, Yun et al. proposed a new reduction agent, p-toluenesulfonyl hydrazine, for the reduction of GO (prGO) [90]. They found that p-toluenesulfonyl hydrazine can effectively remove oxygen-containing functional groups on GO as pristine hydrazine, while new functional groups such as p-toluenesulfonyl were introduced. Because of the incorporation of the bulky p-toluenesulfonyl groups, the dispersion of prGO was better than that of hydrazine reduced GO (hrGO). Therefore, uniform spin-coated prGO films were formed, which were comparable to GO films. In contrast, hrGO films exhibit severe aggregation because of poor dispersion in solvents. The morphologies of GO, hrGO, and prGO are shown in Figure 6d. Interestingly, the conductivity of prGO was similar to that of hrGO regardless of new functional groups being introduced (Figure 6e). When the conditions of reduction were optimized, prGO-based PSCs (P3HT: PCBM) displayed enhanced performance with a PCE of 3.63%, which is significantly higher than that of GO-based devices (PCE = 2.23%) and hrGO-based devices (PCE = 2.83%). Importantly, prGO-based PSCs display significant enhancement in device stability, as shown in Figure 6f. These results indicate a crucial role of functional groups in the performance of GO and rGO based PSCs. Recently, Cheng et al. developed a fluorine-functionalized rGO (F-rGO) employing 2,3,5,6-tetrafluoro-4-(trifluoromethyl)phenylhydrazine hydrazine as a reducing agent and applied F-rGO in PSCs as HTLs [91]. Interestingly, the resulting F-rGO exhibited good dispersion in solvent, a high work function of 5.1 eV, and better conductivity compared to that of GO. The use of F-rGO as HTLs in PSCs yielded better device performance (PCE = 8.6%) compared with that of PEDOT: PSS (PCE = 7.9%) and pristine GO (PCE = 7.8%). Instead of using hydrazine as a reducing agent, Liu et al. improved the conductivity of GO by treating it with fuming sulfuric acid. The authors found that the –OSO_3_H groups were attached to the reduced basel plan of rGO surrounded by edge-functionalized –COOH groups. These functional groups help maintain the solubility of GO-OSO_3_H in the solution while improving the conductivity from 0.004 S m^−1^ to 1.3 S m^–1^, thus improving the PCE of the PSCs (P3HT: PCBM) from 3.34% (GO-based devices) to 4.37% [92].

In addition, GO and rGO can form layer-by-layer films or blending films with other HTLs to improve the overall performance of PSCs in terms of efficiency and stability because the work function of GO can be modulated between the transparent indium tin oxide electrode (ITO) and PEDOT: PSS. The insertion of GO between ITO and conventional HTLs can result in cascade energy levels, which facilitate the charge transport from the active layers to the anode (Figure 6g). Furthermore, GO can protect the ITO glass from corrosion caused by the highly acidic nature of PEDOT: PSS. Therefore, the use of double layers HTLs comprising GO can significantly improve the conversion efficiency and stability of PSCs. For example, Refiqua et al. demonstrated a pronounced enhancement in the performance of PSCs using a GO/PEDOT:PSS double deck as HTLs (PCE = 4.28%). This performance is considerably better than that of devices using individual HTLs such as GO (PCE = 2.77%) and PEDOT: PSS (3.57%) (Figure 6h) [93]. In addition, by using GO/PEDOT: PSS HTLs, Refique et al. discovered that the additional appropriate UVO-treatment (10 min) on the surface of the double layer can further enhance PCE up to 5.24% [94]. However, the PCE of the device dramatically dropped to 2.11% because of the damage to the surface of PEDOT: PSS for more than 15 min of UVO treatment. Interestingly, the lifetime of the PSC that used GO/PEDOT: PSS as HTLs was significantly improved in comparison with that of the controlled device. In specific, the PCE of the GO/PEDOT: PSS based PSC was slightly degraded by 20%, while the PCE of PEDOT: PSS based PSC dropped by 70%, after being kept in air for 240 h. The improvement in device stability was attributed to the GO layer that effectively prevented direct contact between the acidic PEDOT: PSS and the ITO substrate. Besides, GO can also be used as an efficient combination to improve the transport properties of other HTLs. For example, Dang et al. obtained high-performance PSCs with a PCE of 5.1% using GO/MoO_3_ composites as HTLs, which is considerably higher than that of GO-based devices (1.59%) and MoO_3_-based devices (2.5%) [95].

**Figure 7:**
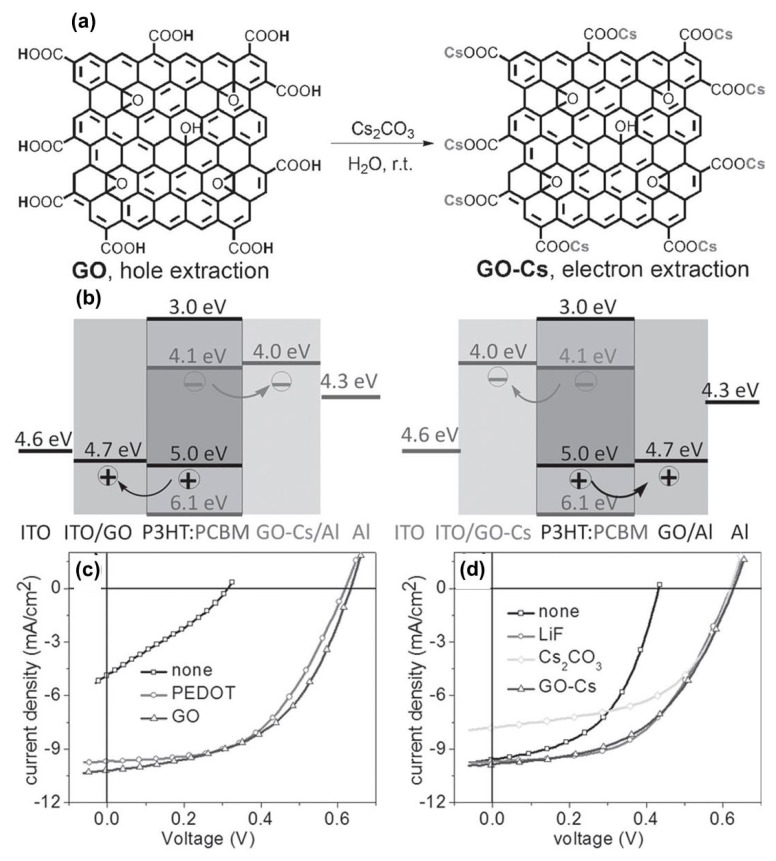
(**a**) Chemical structure and synthetic route of GO and GO-Cs. (**b**) Band diagram of normal and inverted soar cells using GO and GO-Cs as HTLs and electron transport layer (ETLs). (**c**, **d**) Performance of polymer with various HTLs and ETLs. Reproduced with permission [96].

Because the work function of GO is high, its application as ETLs remains questionable. As discussed above, the work function of GO can be reduced through reduction (~4.5 eV); however, it still is not sufficient for the transportation of electrons from PCBM to the anode. To overcome this challenge, Liu et al. proposed a new approach to reduce the work function of GO through doping [96]. Interestingly, the work function of GO was modulated to 4.0 eV after treating with Cs, thus allowing GO to be used as ETLs [96]. The incorporation of Cs into rGO is shown in Figure 7a. The energy levels of normal PSC and inverted PSC using GO-HTLS and CO-Cs-ETLs are shown in Figure 7b. Because of the matching energy levels, PSCs (P3HT: PCBM) exhibited a high PCE of 3.0%, which approached the performance of commercial buffer layers (PEDOT: PSS and LiF) (Figure 7c,d). Furthermore, GO can be applied as ETLs in PSCs by pairing with other low work function metal oxides such as ZnO_2_ and TiO_2_ [97].

### 3.3. Carbon Nanotube Buffer Layers

**Figure 8:**
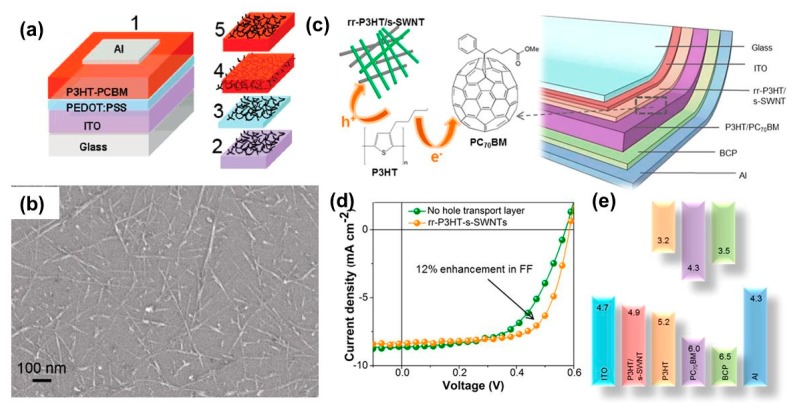
(**a**) Schematic of various devices fabricated for investigation. No. 1 is the control device. Nos. 2–5 represent the layers from the respective devices on which the SWCNT monolayer was dip-coated. For device 4, another active layer was spin-coated after the deposition of the SWNT layer on P3HT/PCBM to effectively embed SWNTs in the P3HT/PCBM phase. (**b**) Monolayer of SWNT dip-coated on an Si substrate from a pristine Dimethylformamide (DMF) suspension. Reproduced with permission [100]. (**c**) Schematic representation of the device architecture used to fabricate solution-processed PSC structures employing rr-P3HT/s-SWNT HTLs. The active layer consists of rr-P3HT/PC70BM, which is sandwiched between rr-P3HT/s-SWNT hole transport and BCP electron transport layers. ITO acts as the anode, whereas Al represents the cathode. (**d**) J–V characteristics of devices A (orange) and B (green) under AM 1.5G illumination with an irradiation intensity of 100 mW cm^−2^. (**e**) Suggested band diagram of the device (ITO/(rr-P3HT/s-SWNT)/(rr-P3HT/PC70BM)/BCP/Al) employing rr-P3HT/s-SWNT nanohybrids as an electron-blocking layer. The energy values are in electron volts. Reproduced with permission [101].

CNTs have been used as a promising candidate for improving the properties of PSCs since their discovery [98,99]. However, the role of CNTs as HTLs was not reported, because of the difficulty in assembling the CNTs into thin films, until 2007 when Chaudhary et al. successfully obtained CNT films by deep coating and applied the films in PSCs [100]. In fact, they studied not only the CNT film as HTLs but also the comprehensive function of CNTs in PSCs. For the investigation, CNTs were assembled in five different positions, as shown in Figure 8a. The SEM image of the assembled CNT film is shown in Figure 8b. The PCE values of PSCs with a configuration from 1 to 5 are 4%, 4.9%, 4.9%, 3.3%, and 0.01%, respectively. Clearly, the device structure numbered two and three performed the best where CNTs acted as HTLs. When CNT was incorporated into the P3HT: PCBM bulk heterojunction (4), the current density and PCE were drastically dropped possibly because of the interference effect of CNTs which decreased the exciton dissociation in the active layers. Instead of fabricating a layer-by-layer structure, Dabera et al. blended SWCNTs with P3HT and used them as HTLs in PTB7:PC_71_BM based PSCs. A high PCE of 7.6% was achieved, which is slightly higher than that of PEDOT: PSS-based devices (PCE ~7.3%) (Figure 8c–e) [101]. Furthermore, a combination of CNTs with other carbon-based HTLs was also reported [102]. For example, Kim et al. found that the addition of a small amount of SWCNT into GO gives rise to a significant enhancement in the conductivity of HTL without affecting morphology [102]. Thus, composite HTLs improved the PCE of PSCs (P3HT: PCBM) from 3.28% to 4.1%. Recently, Zhang et al. found that stand-alone CNTs can work well as HTLs; however, they can be further enhanced via amino functionalization [103]. Typically, the PCE of the PSCs (PCDTBT: PC_71_BM) was improved from 5.7% (pristine CNTs) to 6.97%, which can be attributed to the increase in work function from 5.09 to 5.2 eV.

### 3.4. Graphene Quantum Dots Buffer Layers 

**Figure 9:**
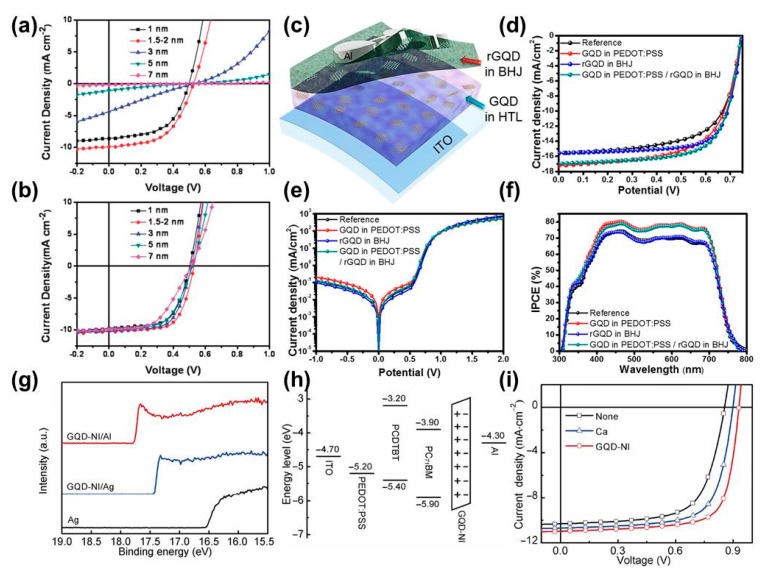
Current density–voltage curves of PSCs using different thicknesses of GO (**a**) or GQDs (**b**) as HTLs. Reproduced with permission [104]. (**c**) Schematic illustration of the device with rGQDs in the BHJ layer and GQDs in HTL (PEDOT: PSS). (**d**) J–V curves, (**e**) dark J–V curves, and (**f**) IPCE of the devices with plain PEDOT: PSS and BHJ (black); PEDOT: PSS with GQDs (red); BHJ with rGQDs (blue); and GQDs and rGQDs added to PEDOT: PSS and BHJ, respectively (green). The concentration ratios of GQDs and rGQDs were 0.4 and 0.02 wt %, respectively. Reproduced with permission [105]. (**g**) Secondary electron cutoff region in ultraviolet photoelectron spectra of GQD-NI interlayer on Al and Ag. (**h**) Energy level diagram of the PSC devices based on the PCDTBT: PC_71_BM active layer with GQD-NI ETLs. (**i**) J–V curves of the PSC devices under AM1.5G illumination with different ETLs using PCDTBT: PC_71_BM as the active layer. Reproduced with permission [106].

Graphene quantum dots exhibit many electronic properties for application as prospective HTLs for high-performance PSCs, such as good conductivity, high work function, and good solubility for a solution-fabrication process. As reported by Li et al., GQD-based PSCs exhibit high performance, which are superior to that of GO as HTLs [104]. Furthermore, the performance of PSCs is less dependent on the thickness of GQDs, as observed in GO-based devices. The device characteristics of PSCs as a function of HTL-thickness are shown in Figure 9a,b. The PCE value of GQD-based devices becomes slightly lower (2.5%) when the thickness is increased to 7 nm, whereas the PCE value of GO-based devices is almost 0% at a GO-thickness of 7 nm. Furthermore, GQDs offered better device stability compared to that of PEDOT: PSS and GO as HTLs. Typically, the PCE of PEDOT: PSS-, GO- and GQDs-based PSCs was measured to be 0%, 34%, and 45% of their initial PCE after exposure to air ambient for 8430 min, without encapsulation. These results suggest that GQDs could be a better alternative than GO for PEDOT: PSS as HTLs.

Similar to the case of GO, functional groups on GQDs enable its high solubility in various solvents. Thus, GQDs can be mixed well with other HTLs such as PEDOT: PSS without severe aggregation. The role of GQDs in GQD/PEDOT: PSS composite HTLs was fully demonstrated in a study conducted by Kim et al. [105]. Typically, they discovered that the addition of GQDs could result in an increase in grain size of the PEDOT: PSS film, possibly because of the interaction between the negatively charged GQDs and positively charged PEDOT polymers. The increase in PEDOT: PSS grains in GQDs/PEDOT: PSS HTLs leads to a significant increase in the current density of the device, thus improving the PCE from 7.52% to 8.17%. The structure and device performance of the PSCs using GQDs/PDOT: PSS as HTLs are shown in Figure 9c–f.

For application as ETLs, several approaches for reducing the work function of GQDs have been developed, including functionalization and doping. For example, Xu et al. functionalized GQDs with ammonium iodide (GQD-NI), showing high electrical conductivity and transparency and appropriate band energy for lowering the barrier between the active layer and cathode [106]. The interfacial characteristics of GQD-NI as ETLs are shown in Figure 9h. As can be seen, the work function of Ag was significantly reduced from 4.8 to 3.8 eV because of the formation of a dipole as the GQD-NI metal interface. In contrast, the GQD-NI modified Al electrode showed an even lower work function of 3.4 eV. The secondary electron cutoff region in the ultraviolet photoelectron spectrum of QDs-NI-modified electrodes is displayed in Figure 9a. The use of GQD-NI as ETLs in PSCs with a structure of ITO/PEDOT: PSS/PCDTBT: PC_71_BM/GQD-NI/Al exhibited a PCE of 7.49%, which is much higher than that of Ca-based ELTs (PCE = 6.72%) (Figure 9i). Similarly, Ding et al. functionalized tetramethylammonium at the edge of GQDs (GQDs-TMA). The functional groups on GQDs acted as an interfacial dipole for decreasing the work function of the cathode, thus improving device performance [107]. Typically, the performance GQDs-TMA-based devices (PCE = 7.01%) is superior to that of other ELTs including Ca, Lif, and ZnO (PCE < 6.5%). In addition to GQDs, CQDs as ETLs in PSCs have also been demonstrated. For example, Yan et al. synthesized CQDs using the chemical vapor deposition method and used them as ETLs in PSCs [108]. The as synthesized CQDs showed an average diameter of 3.5 nm with hydrophobic –CH_3_ terminal groups and a bandgap of 3.5 eV (−7 eV/−3.84 eV). The CQD-based devices showed similar PCEs in comparison to that of LiF-based devices; however, the stability was significantly improved. Instead of using bare CQDs, Wang et al. used N,S doped quantum dots in PSCs as the additive for ZnO-ETLs to boost the PCE of solar cells (PTB7-Th:PC_71_BM) from 4.67% (pristine ZnO) to 9.29% [109]. These results indicate that either GQDs or CQDs can be used as efficient buffer layers in PSCs; however, proper treatment or functionalization is required.

### 3.5. Graphitic Carbon Nitrides

**Figure 10:**
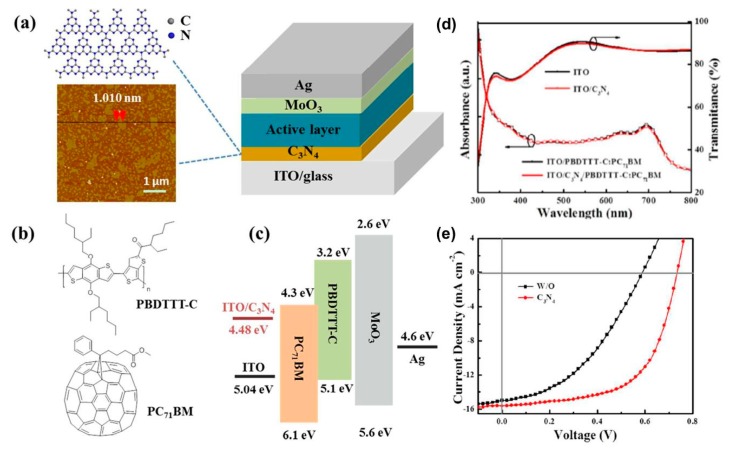
(**a**) Device configuration of the inverted PSCs using C_3_N_4_ as cathode interfacial layers. The left images show the schematic structure of C_3_N_4_ and the AFM topography image of C_3_N_4_ nanosheets on mica substrates. (**b**) Chemical structures of donor material PBDTTT-C and acceptor PC71BM. (**c**) Schematic energy level diagram of the inverted PSCs. (**d**) Transmittance spectra of ITO and ITO/ C_3_N_4_ and optical absorption spectra of ITO/PBDTTT-C: PC_71_BM and ITO/ C_3_N_4_/PBDTTT-C: PC_71_BM. (**e**) J–V characteristics of inverted PSCs with and without C_3_N_4_ as ETL. Reproduced with permission [112].

Generally, g-C_3_N_4_ is used in PSCs as an acceptor associated with organic donors such as P3HT, resulting in a significant improvement in the open-circuit voltage (V_oc_) [110]. Chen et al. demonstrated that the incorporation of g-C_3_N_4_ into bulk heterojunction PSCs could improve their PCE by more than 10% [111]. The use of g-C_3_N_4_ as a buffer layer in PSCs has also been reported [112,113]. The device structure and energy band diagram of inverted PSCs that use g-C_3_N_4_ as ETLs are shown in Figure 10a–c [112]. The work function of g-C_3_N_4_ lies between the lowest unoccupied molecular orbital (LUMO) of PC_71_PM and ITO; thus, it can facilitate the transportation of electrons from the active layer to the anode. In addition, the C_3_N_4_ coated ITO showed transmittance similar to that of the bare ITO substrate (Figure 10d), indicating that it can be used as a transparent buffer layer in PSCs. From Figure 10e, it is clear that the use of g-C_3_N_4_ as ETLs can effectively increase the overall device performance. Specifically, the PCE of PSCs was increased from 3.67% (without ETLs) to 6.4%. This result indicates that g-C_3_N_4_ is a promising candidate as ETL; however, more investigations are required. Furthermore, to our best knowledge, the application of g-C_3_N_4_ as HTLs in PSCs has not been reported yet; thus, there is still room for investigation for future development.

## 4. Conclusions and Outlooks

In this review, we have rationally highlighted and discussed the recent progress in the development of carbon-based materials as buffer layers in PSCs. We also highlighted strategies to improve the charge-transfer properties of carbon-based materials, such as thermal treatment, chemical treatment, functionalization, doping, and compositional engineering, thus improving device efficiency. The performance of carbon-based HTLs in PSCs was found to be comparable to that of commercial PEDOT: PSS. Importantly, the lifetime of devices has been significantly improved. Furthermore, carbon-based materials have also proven to be efficient ETLs. Typically, GO and GQDs were demonstrated to be compatible with either ETLs or HTLs in PSCs. All-carbon buffer layers-based devices have also been reported with remarkable high performance and stability, suggesting that carbon-based buffer layers are essential for the future development of PSCs, which require more attention and investigation in the near future. The next stage of research on carbon-based buffer layers should focus on (1) developing large-scale synthesis methods for carbon materials via low-temperature solution processes, (2) optimizing the energy level and conductivity of HTLs, and (3) developing a roll-to-roll deposition process to control the thickness and uniformity of the products, thus targeting industrialization.

## References

[1] Blakers A., Zin N., McIntosh K.R., Fong K. (2013). High Efficiency Silicon Solar Cells. Energy Procedia.

[2] Kumar A., Bieri M., Reindl T., Aberle A.G. (2017). Economic Viability Analysis of Silicon Solar Cell Manufacturing: Al-BSF versus PERC. Energy Procedia.

[3] Pan Z., Rao H., Mora-Seró I., Bisquert J., Zhong X. (2018). Quantum dot-sensitized solar cells. Chem. Soc. Rev..

[4] Ganesan A.A., Houtepen A.J., Crisp R.W. (2018). Quantum Dot Solar Cells: Small Beginnings Have Large Impacts. Appl. Sci..

[5] Ahmad W., He J., Liu Z., Xu K., Chen Z., Yang X., Li D., Xia Y., Zhang J., Chen C. (2019). Lead Selenide (PbSe) Colloidal Quantum Dot Solar Cells with >10% Efficiency. Adv. Mater..

[6] Gong J., Sumathy K., Qiao Q., Zhou Z. (2017). Review on dye-sensitized solar cells (DSSCs): Advanced techniques and research trends. Renew. Sustain. Energ. Rev..

[7] Upadhyaya H.M., Senthilarasu S., Hsu M.-H., Kumar D.K. (2013). Recent progress and the status of dye-sensitised solar cell (DSSC) technology with state-of-the-art conversion efficiencies. Sol. Energy Mater. Sol. Cells.

[8] Hou W., Xiao Y., Han G., Lin J.-Y. (2019). The Applications of Polymers in Solar Cells: A Review. Polymers.

[9] Sun H., Chen F., Chen Z.-K. (2019). Recent progress on non-fullerene acceptors for organic photovoltaics. Mater. Today.

[10] Wang G., Melkonyan F.S., Facchetti A., Marks T.J. (2019). All-Polymer Solar Cells: Recent Progress, Challenges, and Prospects. Angew. Chem. Int. Ed..

[11] Liu Z., Zhang L., Shao M., Wu Y., Zeng D., Cai X., Duan J., Zhang X., Gao X. (2018). Fine-Tuning the Quasi-3D Geometry: Enabling Efficient Nonfullerene Organic Solar Cells Based on Perylene Diimides. ACS Appl. Mater. Interfaces.

[12] Liu Z., Wu Y., Zhang Q., Gao X. (2016). Non-fullerene small molecule acceptors based on perylene diimides. J. Mater. Chem. A.

[13] Ramanujam J., Singh U.P. (2017). Copper indium gallium selenide based solar cells—A review. Energy Environ. Sci..

[14] Tai Q., Tang K.-C., Yan F. (2019). Recent progress of inorganic perovskite solar cells. Energy Environ. Sci..

[15] Assadi M.K., Bakhoda S., Saidur R., Hanaei H. (2018). Recent progress in perovskite solar cells. Renew. Sustain. Energ. Rev..

[16] Shi Z., Jayatissa A.H. (2018). Perovskites-Based Solar Cells: A Review of Recent Progress, Materials and Processing Methods. Materials.

[17] Espinosa N., Dam H.F., Tanenbaum D.M., Andreasen J.W., Jørgensen M., Krebs F.C. (2011). Roll-to-Roll Processing of Inverted Polymer Solar Cells using Hydrated Vanadium(V)Oxide as a PEDOT:PSS Replacement. Materials.

[18] Gu X., Zhou Y., Gu K., Kurosawa T., Guo Y., Li Y., Lin H., Schroeder B.C., Yan H., Molina-Lopez F. (2017). Roll-to-Roll Printed Large-Area All-Polymer Solar Cells with 5% Efficiency Based on a Low Crystallinity Conjugated Polymer Blend. Adv. Energy Mater..

[19] Sun C., Pan F., Bin H., Zhang J., Xue L., Qiu B., Wei Z., Zhang Z.-G., Li Y. (2018). A low cost and high performance polymer donor material for polymer solar cells. Nat. Commun..

[20] Lu S., Sun Y., Ren K., Liu K., Wang Z., Qu S. (2018). Recent Development in ITO-free Flexible Polymer Solar Cells. Polymers.

[21] Cheng P., Zhan X. (2016). Stability of organic solar cells: Challenges and strategies. Chem. Soc. Rev..

[22] Mirsafaei M., Fallahpour A.H., Lugli P., Rubahn H.-G., Adam J., Madsen M. (2017). The influence of electrical effects on device performance of organic solar cells with nano-structured electrodes. Sci. Rep..

[23] Po R., Carbonera C., Bernardi A., Camaioni N. (2011). The role of buffer layers in polymer solar cells. Energy Environ. Sci..

[24] Kim Y.-H., Lee S.-H., Noh J., Han S.-H. (2006). Performance and stability of electroluminescent device with self-assembled layers of poly(3,4-ethylenedioxythiophene)–poly(styrenesulfonate) and polyelectrolytes. Thin Solid Films.

[25] Jørgensen M., Norrman K., Krebs F.C. (2008). Stability/degradation of polymer solar cells. Sol. Energy Mater. Sol. Cells.

[26] Kawano K., Pacios R., Poplavskyy D., Nelson J., Bradley D.D.C., Durrant J.R. (2006). Degradation of organic solar cells due to air exposure. Sol. Energy Mater. Sol. Cells.

[27] Dubey R., Guruviah V. (2019). Review of carbon-based electrode materials for supercapacitor energy storage. Ionics.

[28] Najib S., Erdem E. (2019). Current progress achieved in novel materials for supercapacitor electrodes: Mini review. Nanoscale Adv..

[29] Zhang L., Wang Y., Niu Z., Chen J. (2019). Advanced nanostructured carbon-based materials for rechargeable lithium-sulfur batteries. Carbon N. Y..

[30] Roselin L.S., Juang R.-S., Hsieh C.-T., Sagadevan S., Umar A., Selvin R., Hegazy H.H. (2019). Recent Advances and Perspectives of Carbon-Based Nanostructures as Anode Materials for Li-ion Batteries. Materials.

[31] Lam E., Luong J.H.T. (2014). Carbon Materials as Catalyst Supports and Catalysts in the Transformation of Biomass to Fuels and Chemicals. ACS Catal..

[32] Anthonysamy S.B.I., Afandi S.B., Khavarian M., Mohamed A.R.B. (2018). A review of carbon-based and non-carbon-based catalyst supports for the selective catalytic reduction of nitric oxide. Beilstein J. Nanotechnol..

[33] Meng F., Liu A., Gao L., Cao J., Yan Y., Wang N., Fan M., Wei G., Ma T. (2019). Current progress in interfacial engineering of carbon-based perovskite solar cells. J. Mater. Chem. A.

[34] Ferguson V., Silva S.R.P., Zhang W. (2019). Carbon Materials in Perovskite Solar Cells: Prospects and Future Challenges. Energy Environ. Mater..

[35] Guo C.X., Guai G.H., Li C.M. (2011). Graphene Based Materials: Enhancing Solar Energy Harvesting. Adv. Energy Mater..

[36] Wang G., Wang B., Park J., Wang Y., Sun B., Yao J. (2009). Highly efficient and large-scale synthesis of graphene by electrolytic exfoliation. Carbon N. Y..

[37] Liu J., Shao M., Chen X., Yu W., Liu X., Qian Y. (2003). Large-Scale Synthesis of Carbon Nanotubes by an Ethanol Thermal Reduction Process. J. Am. Chem. Soc..

[38] Fang H.-B., Luo Y., Zheng Y.-Z., Ma W., Tao X. (2016). Facile Large-Scale Synthesis of Urea-Derived Porous Graphitic Carbon Nitride with Extraordinary Visible-Light Spectrum Photodegradation. Ind. Eng. Chem. Res..

[39] Dong L., Yang J., Chhowalla M., Loh K.P. (2017). Synthesis and reduction of large sized graphene oxide sheets. Chem. Soc. Rev..

[40] Wang Z., Yu J., Zhang X., Li N., Liu B., Li Y., Wang Y., Wang W., Li Y., Zhang L. (2016). Large-Scale and Controllable Synthesis of Graphene Quantum Dots from Rice Husk Biomass: A Comprehensive Utilization Strategy. ACS Appl. Mater. Interfaces.

[41] Yang G., Li L., Lee W.B., Ng M.C. (2018). Structure of graphene and its disorders: A review. Sci. Technol. Adv. Mater..

[42] Xu Y., Cao H., Xue Y., Li B., Cai W. (2018). Liquid-Phase Exfoliation of Graphene: An Overview on Exfoliation Media, Techniques, and Challenges. Nanomaterials.

[43] Yi M., Shen Z. (2015). A review on mechanical exfoliation for the scalable production of graphene. J. Mater. Chem. A.

[44] Zhang Y., Zhang L., Zhou C. (2013). Review of Chemical Vapor Deposition of Graphene and Related Applications. Acc. Chem. Res..

[45] Marinho B., Ghislandi M., Tkalya E., Koning C.E., de With G. (2012). Electrical conductivity of compacts of graphene, multi-wall carbon nanotubes, carbon black, and graphite powder. Powder Technol..

[46] Sang M., Shin J., Kim K., Yu K.J. (2019). Electronic and Thermal Properties of Graphene and Recent Advances in Graphene Based Electronics Applications. Nanomaterials.

[47] Balandin A.A., Ghosh S., Bao W., Calizo I., Teweldebrhan D., Miao F., Lau C.N. (2008). Superior Thermal Conductivity of Single-Layer Graphene. Nano Lett..

[48] Zhu S.-E., Yuan S., Janssen G.C.A.M. (2014). Optical transmittance of multilayer graphene. EPL.

[49] Zhu Y., Murali S., Cai W., Li X., Suk J.W., Potts J.R., Ruoff R.S. (2010). Graphene and Graphene Oxide: Synthesis, Properties, and Applications. Adv. Mater..

[50] Lee C., Wei X., Kysar J.W., Hone J. (2008). Measurement of the Elastic Properties and Intrinsic Strength of Monolayer Graphene. Science.

[51] Han T.-H., Kim H., Kwon S.-J., Lee T.-W. (2017). Graphene-based flexible electronic devices. Mater. Sci. Eng. R Rep..

[52] Yadav A., Upadhyaya A., Gupta S.K., Verma A.S., Singh Negi C.M. (2018). Solution processed graphene as electron transport layer for bulk heterojunction based devices. Supperlattices Microst..

[53] Biccari F., Gabelloni F., Burzi E., Gurioli M., Pescetelli S., Agresti A., Del Rio Castillo A.E., Ansaldo A., Kymakis E., Bonaccorso F. (2017). Graphene-Based Electron Transport Layers in Perovskite Solar Cells: A Step-Up for an Efficient Carrier Collection. Adv. Energy Mater..

[54] Chen D., Feng H., Li J. (2012). Graphene Oxide: Preparation, Functionalization, and Electrochemical Applications. Chem. Rev..

[55] Singh R.K., Kumar R., Singh D.P. (2016). Graphene oxide: Strategies for synthesis, reduction and frontier applications. RSC Adv..

[56] Mao S., Pu H., Chen J. (2012). Graphene oxide and its reduction: Modeling and experimental progress. RSC Adv..

[57] Erickson K., Erni R., Lee Z., Alem N., Gannett W., Zettl A. (2010). Determination of the Local Chemical Structure of Graphene Oxide and Reduced Graphene Oxide. Adv. Mater..

[58] Yun-Chieh Y., Di-Yan W., Chun-Wei C. Work function evolution of graphene oxide by utilizing hydrothermal treatment. Proceedings of the 8th International Vacuum Electron Sources Conference and Nanocarbon.

[59] Aqel A., El-Nour K.M.M.A., Ammar R.A.A., Al-Warthan A. (2012). Carbon nanotubes, science and technology part (I) structure, synthesis and characterisation. Arab. J. Chem..

[60] Rahman G., Najaf Z., Mehmood A., Bilal S., Shah A.u.H.A., Mian S.A., Ali G. (2019). An Overview of the Recent Progress in the Synthesis and Applications of Carbon Nanotubes. C J. Carbon Res..

[61] Tang Z.K., Zhang L., Wang N., Zhang X.X., Wen G.H., Li G.D., Wang J.N., Chan C.T., Sheng P. (2001). Superconductivity in 4 Angstrom Single-Walled Carbon Nanotubes. Science.

[62] Dresselhaus M.S., Dresselhaus G., Saito R. (1995). Physics of carbon nanotubes. Carbon N. Y..

[63] Zhang F., Hou P.-X., Liu C., Cheng H.-M. (2016). Epitaxial growth of single-wall carbon nanotubes. Carbon N. Y..

[64] Eatemadi A., Daraee H., Karimkhanloo H., Kouhi M., Zarghami N., Akbarzadeh A., Abasi M., Hanifehpour Y., Joo S.W. (2014). Carbon nanotubes: Properties, synthesis, purification, and medical applications. Nanoscale Res. Lett..

[65] Zhou Y., Azumi R. (2016). Carbon nanotube based transparent conductive films: Progress, challenges, and perspectives. Sci. Technol. Adv. Mater..

[66] Shiraishi M., Ata M. (2001). Work function of carbon nanotubes. Carbon N. Y..

[67] Ding C., Zhu A., Tian Y. (2014). Functional Surface Engineering of C-Dots for Fluorescent Biosensing and in Vivo Bioimaging. Acc. Chem. Res..

[68] Roy P., Chen P.-C., Periasamy A.P., Chen Y.-N., Chang H.-T. (2015). Photoluminescent carbon nanodots: Synthesis, physicochemical properties and analytical applications. Mater. Today.

[69] Namdari P., Negahdari B., Eatemadi A. (2017). Synthesis, properties and biomedical applications of carbon-based quantum dots: An updated review. Biomed. Pharm..

[70] Zhu S., Song Y., Wang J., Wan H., Zhang Y., Ning Y., Yang B. (2017). Photoluminescence mechanism in graphene quantum dots: Quantum confinement effect and surface/edge state. Nano Today.

[71] Zhang L., Ding Z.C., Tong T., Liu J. (2017). Tuning the work functions of graphene quantum dot-modified electrodes for polymer solar cell applications. Nanoscale.

[72] Miller T.S., Jorge A.B., Suter T.M., Sella A., Corà F., McMillan P.F. (2017). Carbon nitrides: Synthesis and characterization of a new class of functional materials. Phys. Chem. Chem. Phys..

[73] Wang A., Wang C., Fu L., Wong-Ng W., Lan Y. (2017). Recent Advances of Graphitic Carbon Nitride-Based Structures and Applications in Catalyst, Sensing, Imaging, and LEDs. Nano-Micro Lett..

[74] Liao G., Gong Y., Zhang L., Gao H., Yang G.-J., Fang B. (2019). Semiconductor polymeric graphitic carbon nitride photocatalysts: The “holy grail” for the photocatalytic hydrogen evolution reaction under visible light. Energy Environ. Sci..

[75] Lv H., Hu H., Cui C., Lin P., Wang P., Wang H., Xu L., Pan J., Li C. (2017). Enhanced performance of dye-sensitized solar cells with layered structure graphitic carbon nitride and reduced graphene oxide modified TiO_2_ photoanodes. Appl. Surf. Sci..

[76] Naseri A., Samadi M., Pourjavadi A., Moshfegh A.Z., Ramakrishna S. (2017). Graphitic carbon nitride (g-C_3_N_4_)-based photocatalysts for solar hydrogen generation: Recent advances and future development directions. J. Mater. Chem. A.

[77] Mishra A., Mehta A., Basu S., Shetti N.P., Reddy K.R., Aminabhavi T.M. (2019). Graphitic carbon nitride (g-C_3N4_)-based metal-free photocatalysts for water splitting: A review. Carbon N. Y..

[78] Cao S., Yu J. (2014). g-C_3_N_4_-Based Photocatalysts for Hydrogen Generation. J. Phys. Chem. Lett..

[79] Wang M., Ma F., Wang Z., Hu D., Xu X., Hao X. (2018). Graphitic carbon nitride, a saturable absorber material for the visible waveband. Photonics Res..

[80] Zhu B., Zhang J., Jiang C., Cheng B., Yu J. (2017). First principle investigation of halogen-doped monolayer g-C_3_N_4_ photocatalyst. Appl. Catal. B Environ..

[81] Dang Y., Wang Y., Shen S., Huang S., Qu X., Pang Y., Silva S.R.P., Kang B., Lu G. (2019). Solution processed hybrid Graphene-MoO_3_ hole transport layers for improved performance of organic solar cells. Org. Electron..

[82] Iakobson O.D., Gribkova O.L., Tameev A.R., Nekrasov A.A., Saranin D.S., Di Carlo A. (2018). Graphene nanosheet/polyaniline composite for transparent hole transporting layer. J. Ind. Eng. Chem..

[83] Li S.-S., Tu K.-H., Lin C.-C., Chen C.-W., Chhowalla M. (2010). Solution-Processable Graphene Oxide as an Efficient Hole Transport Layer in Polymer Solar Cells. ACS Nano.

[84] Rafique S., Abdullah S.M., Iqbal J., Jilani A., Vattamkandathil S., Iwamoto M. (2018). Moderately reduced graphene oxide via UV-ozone treatment as hole transport layer for high efficiency organic solar cells. Org. Electron..

[85] Xia Y., Pan Y., Zhang H., Qiu J., Zheng Y., Chen Y., Huang W. (2017). Graphene Oxide by UV-Ozone Treatment as an Efficient Hole Extraction Layer for Highly Efficient and Stable Polymer Solar Cells. ACS Appl. Mater. Interfaces.

[86] Jeon Y.-J., Yun J.-M., Kim D.-Y., Na S.-I., Kim S.-S. (2012). High-performance polymer solar cells with moderately reduced graphene oxide as an efficient hole transporting layer. Sol. Energy Mater. Sol. Cells.

[87] Liu X., Kim H., Guo L.J. (2013). Optimization of thermally reduced graphene oxide for an efficient hole transport layer in polymer solar cells. Org. Electron..

[88] Murray I.P., Lou S.J., Cote L.J., Loser S., Kadleck C.J., Xu T., Szarko J.M., Rolczynski B.S., Johns J.E., Huang J. (2011). Graphene Oxide Interlayers for Robust, High-Efficiency Organic Photovoltaics. J. Phys. Chem. Lett..

[89] Kwon S.-N., Jung C.-H., Na S.-I. (2016). Electron-beam-induced reduced graphene oxide as an alternative hole-transporting interfacial layer for high-performance and reliable polymer solar cells. Org. Electron..

[90] Yun J.-M., Yeo J.-S., Kim J., Jeong H.-G., Kim D.-Y., Noh Y.-J., Kim S.-S., Ku B.-C., Na S.-I. (2011). Solution-Processable Reduced Graphene Oxide as a Novel Alternative to PEDOT:PSS Hole Transport Layers for Highly Efficient and Stable Polymer Solar Cells. Adv. Mater..

[91] Cheng X., Long J., Wu R., Huang L., Tan L., Chen L., Chen Y. (2017). Fluorinated Reduced Graphene Oxide as an Efficient Hole-Transport Layer for Efficient and Stable Polymer Solar Cells. ACS Omega.

[92] Liu J., Xue Y., Dai L. (2012). Sulfated Graphene Oxide as a Hole-Extraction Layer in High-Performance Polymer Solar Cells. J. Phys. Chem. Lett..

[93] Rafique S., Abdullah S.M., Shahid M.M., Ansari M.O., Sulaiman K. (2017). Significantly improved photovoltaic performance in polymer bulk heterojunction solar cells with graphene oxide /PEDOT:PSS double decked hole transport layer. Sci. Rep..

[94] Rafique S., Roslan N.A., Abdullah S.M., Li L., Supangat A., Jilani A., Iwamoto M. (2019). UV-ozone treated graphene oxide/PEDOT:PSS bilayer as a novel hole transport layer in highly efficient and stable organic solar cells. Org. Electron..

[95] Aatif M., Patel J., Sharma A., Chauhan M., Kumar G., Pal P., Chand S., Tripathi B., Pandey M.K., Tiwari J.P. (2019). Graphene oxide-molybdenum oxide composite with improved hole transport in bulk heterojunction solar cells. AIP Adv..

[96] Liu J., Xue Y., Gao Y., Yu D., Durstock M., Dai L. (2012). Hole and Electron Extraction Layers Based on Graphene Oxide Derivatives for High-Performance Bulk Heterojunction Solar Cells. Adv. Mater..

[97] Jayawardena K.D.G.I., Rhodes R., Gandhi K.K., Prabhath M.R.R., Dabera G.D.M.R., Beliatis M.J., Rozanski L.J., Henley S.J., Silva S.R.P. (2013). Solution processed reduced graphene oxide/metal oxide hybrid electron transport layers for highly efficient polymer solar cells. J. Mater. Chem. A.

[98] Kymakis E., Alexandrou I., Amaratunga G.A.J. (2003). High open-circuit voltage photovoltaic devices from carbon-nanotube-polymer composites. J. Appl. Phys..

[99] Rowell M.W., Topinka M.A., McGehee M.D., Prall H.-J., Dennler G., Sariciftci N.S., Hu L., Gruner G. (2006). Organic solar cells with carbon nanotube network electrodes. Appl. Phys. Lett..

[100] Chaudhary S., Lu H., Müller A.M., Bardeen C.J., Ozkan M. (2007). Hierarchical Placement and Associated Optoelectronic Impact of Carbon Nanotubes in Polymer-Fullerene Solar Cells. Nano Lett..

[101] Dabera G.D.M.R., Jayawardena K.D.G.I., Prabhath M.R.R., Yahya I., Tan Y.Y., Nismy N.A., Shiozawa H., Sauer M., Ruiz-Soria G., Ayala P. (2013). Hybrid Carbon Nanotube Networks as Efficient Hole Extraction Layers for Organic Photovoltaics. ACS Nano.

[102] Kim J., Tung V.C., Huang J. (2011). Water Processable Graphene Oxide:Single Walled Carbon Nanotube Composite as Anode Modifier for Polymer Solar Cells. Adv. Energy Mater..

[103] Zhang X., Sun S., Liu X. (2019). Amino functionalized carbon nanotubes as hole transport layer for high performance polymer solar cells. Inorg. Chem. Commun..

[104] Li M., Ni W., Kan B., Wan X., Zhang L., Zhang Q., Long G., Zuo Y., Chen Y. (2013). Graphene quantum dots as the hole transport layer material for high-performance organic solar cells. Phys. Chem. Chem. Phys..

[105] Kim J.K., Kim S.J., Park M.J., Bae S., Cho S.-P., Du Q.G., Wang D.H., Park J.H., Hong B.H. (2015). Surface-Engineered Graphene Quantum Dots Incorporated into Polymer Layers for High Performance Organic Photovoltaics. Sci. Rep..

[106] Xu H., Zhang L., Ding Z., Hu J., Liu J., Liu Y. (2018). Edge-functionalized graphene quantum dots as a thickness-insensitive cathode interlayer for polymer solar cells. Nano Res..

[107] Ding Z., Miao Z., Xie Z., Liu J. (2016). Functionalized graphene quantum dots as a novel cathode interlayer of polymer solar cells. J. Mater. Chem. A.

[108] Yan L., Yang Y., Ma C.-Q., Liu X., Wang H., Xu B. (2016). Synthesis of carbon quantum dots by chemical vapor deposition approach for use in polymer solar cell as the electrode buffer layer. Carbon N. Y..

[109] Wang Y., Yan L., Ji G., Wang C., Gu H., Luo Q., Chen Q., Chen L., Yang Y., Ma C.-Q. (2019). Synthesis of N,S-Doped Carbon Quantum Dots for Use in Organic Solar Cells as the ZnO Modifier To Eliminate the Light-Soaking Effect. ACS Appl. Mater. Interfaces.

[110] Xu J., Brenner T.J.K., Chabanne L., Neher D., Antonietti M., Shalom M. (2014). Liquid-Based Growth of Polymeric Carbon Nitride Layers and Their Use in a Mesostructured Polymer Solar Cell with Voc Exceeding 1 V. J. Am. Chem. Soc..

[111] Chen X., Liu Q., Wu Q., Du P., Zhu J., Dai S., Yang S. (2016). Incorporating Graphitic Carbon Nitride (g-C_3_N_4_) Quantum Dots into Bulk-Heterojunction Polymer Solar Cells Leads to Efficiency Enhancement. Adv. Funct. Mater..

[112] Zhou L., Xu Y., Yu W., Guo X., Yu S., Zhang J., Li C. (2016). Ultrathin two-dimensional graphitic carbon nitride as a solution-processed cathode interfacial layer for inverted polymer solar cells. J. Mater. Chem. A.

[113] Soh M.F., Noh M.F.M., Mohamed N.A., Safaei J., Rosli N.N., Lim E.L., Yap C.C., Teridi M.A.M. (2019). Incorporation of g-C_3_N_4_/Ag dopant in TiO_2_ as electron transport layer for organic solar cells. Mater. Lett..

